# Chinese medicine *Ginseng* and *Astragalus* granules ameliorate autoimmune diabetes by upregulating both CD4+FoxP3+ and CD8+CD122+PD1+ regulatory T cells

**DOI:** 10.18632/oncotarget.18732

**Published:** 2017-06-27

**Authors:** Yeshu Wang, Qingfeng Xie, Chun-Ling Liang, Qiaohuang Zeng, Zhenhua Dai

**Affiliations:** ^1^ Graduate School, Guangzhou University of Chinese Medicine, Guangzhou, Guangdong, P.R. China; ^2^ Center for Regenerative and Translational Medicine, Guangdong Provincial Academy of Chinese Medical Sciences, Guangdong Provincial Hospital of Chinese Medicine, Guangzhou, Guangdong, P.R. China; ^3^ Section of Immunology, Guangdong Provincial Academy of Chinese Medical Sciences, Guangzhou, Guangdong, P.R. China

**Keywords:** autoimmunity, type 1 diabetes, regulatory T cells, Chinese medicine

## Abstract

Type 1 diabetes mellitus (T1DM) is an autoimmune disease mainly mediated by effector T cells that are activated by autoantigen, thereby resulting in the destruction of pancreatic islets and deficiency of insulin. Cyclosporine is widely used as an immunosuppressant that suppresses autoimmunity in clinic. However, continuous treatments with conventional immunosuppressive drugs may cause severe side effects. Therefore it is important to seek alternative medicine. Chinese medicine *Ginseng* and *Astragalus* granule (GAG) was used to successfully treat type 2 diabetes mellitus in clinic in China. Here we found that GAG ameliorated T1DM in autoimmune NOD mice by increasing the level of insulin and reducing the level of blood glucose. Treatments with both GAG and CsA further decreased the blood glucose level. Moreover, GAG increased both CD4+FoxP3+ and CD8+CD122+PD-1+ Treg numbers in both spleens and lymph nodes of NOD mice. In particular, GAG could reverse a decline in CD4+FoxP3+ Tregs resulted from CsA treatments. The percentage of effector/memory CD8+ T cells (CD44^high^CD62L^low^) was significantly reduced by GAG, especially in the presence of low-doses of CsA. Histopathology also showed that GAG attenuated cellular infiltration and lowered CD3+ T cell numbers around and in islets. Thus, we demonstrated that GAG ameliorated autoimmune T1DM by upregulating both CD4+FoxP3+ and CD8+CD122+PD-1+ Tregs while GAG synergized with CsA to further suppress autoimmunity and T1DM by reversing the decline in CD4+FoxP3+ Tregs resulted from CsA treatments. This study may have important clinical implications for the treatment of T1DM using traditional Chinese medicine.

## INTRODUCTION

Type 1 diabetes mellitus (T1DM) is an autoimmune disease mainly mediated by effector T lymphocytes that are activated by an autoantigen and then destroy pancreatic islets, thereby resulting in the deficiency of insulin production [1, 2]. Regulatory T cells (Treg), a subset of T cells that can suppress autoimmune reactions, play a crucial role in sustaining autoimmune tolerance and preventing autoimmune diseases. However, its number and function were shown to be quite low in some autoimmune diseases, such as T1DM [3–8]. On the other hand, effector T cells play an important role in both humoral and cellular immune responses as traditionally aggressive T cells. An imbalance between Tregs and autoreactive effector T cells in favor of the effector cells could result in the onset of various autoimmune diseases.

Treatments of T1DM are dependent on long-term injection of insulin when pancreatic islets are totally destroyed although immunosuppressive agents could reverse T1DM during the early stage of the disease. However, it may require continuous treatments with a conventional immunosuppressant that usually causes side effects. Traditional Chinese medicine Ginseng and Astragalus granules (GAG), mainly composed of Ginseng and Astragalus, is widely used to treat type 2 diabetes mellitus in China [9, 10]. GAG contains 10 herbs, including Ginsenosides, Schisandra, Astragalus, Yam, Radix rehmanniae, Ophiopogon japonicus, Poria, Radix trichosanthis, Rhizoma alismatis and Chinese wolfberry. Previous studies have also demonstrated that GAG significantly ameliorates hyperglycemia, increases blood insulin levels, and improves the glucose tolerance through enhancing islet cell function [11]. However, it is unknown whether GAG can be utilized to treat T1DM and suppress autoimmune responsiveness.

Here, we examined the effects of Chinese medicine GAG on the onset of T1DM in female NOD mice. We found that administration of GAG decreased the level of blood glucose but elevated blood insulin level of NOD mice. Importantly, administering both GAG and low doses of CsA further ameliorated autoimmune T1DM compared with treatments with either GAG or CsA alone. Moreover, GAG augmented both CD4+FoxP3+ and CD8+CD122+PD1+ Tregs in spleens and lymph nodes of NOD mice. In particular, it was capable of increasing CD4+FoxP3+ Tregs even in the presence of CsA.

## RESULTS

### GAG significantly decreases the level of blood glucose in T1DM-prone NOD mice

**Figure 1:**
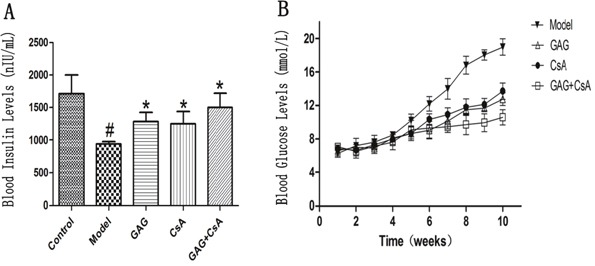
GAG significantly increases blood insulin but decreases blood glucose level Three months-old female NOD mice were treated with GAG and/or CsA for eight weeks, and then the blood insulin level was detected by ELISA **(A)**. The values of blood glucose were also detected using Opitima Xceed and Test Strips weekly for ten weeks after the first treatment with GAG **(B)**. Data are presented as Mean ± SD (n = 4 - 5 mice/group). One representative of two independent experiments is shown (# represents comparison with Control; and * represents comparison with Model, both p<0.05).

We determined whether GAG, which was widely used to treat type 2 diabetes mellitus, could also have an impact on type 1 diabetes mellitus (T1DM). GAG was administered by oral gavage at 3g/kg/d while CsA was injected intraperitoneally at 7.5 mg/kg/d to three-months old female NOD mice daily for eight weeks. As shown in Figure 1A, we found that GAG alone obviously increased the level of blood insulin eight weeks after the treatments. Similar findings were seen at the time point of four weeks (Data not shown). On the other hand, GAG alone decreased blood glucose level while CsA alone also did the same (Figure 1B). However, the best result was brought by the combination of GAG and CsA, which further reduced the blood glucose level compared with either GAG alone or CsA alone.

### GAG induces both CD4+FoxP3+ and CD8+CD122+PD-1+ Tregs even in the presence of low-doses of CsA

**Figure 2:**
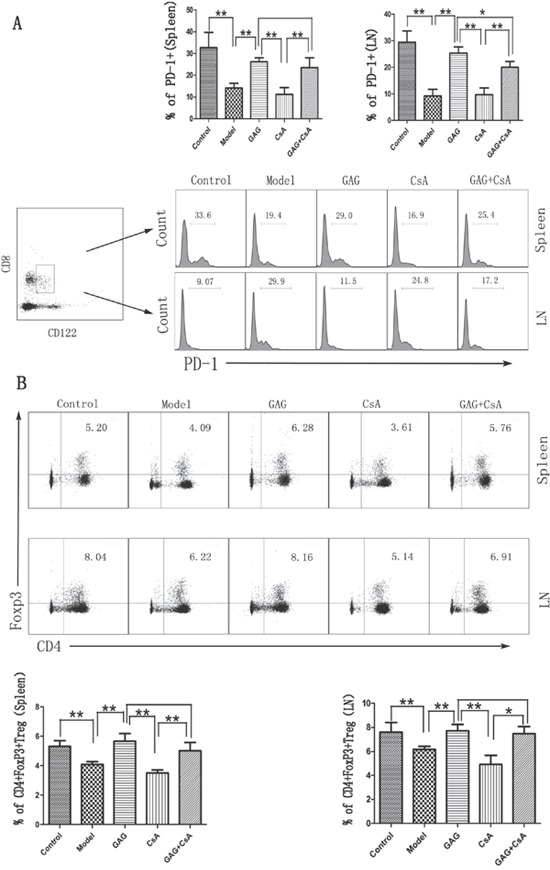
GAG induces both CD8+CD122+PD-1+ and CD4+FoxP3+ Tregs in NOD mice Spleen and lymph node cells were isolated from female NOD mice eight weeks after treatments with GAG and/or CsA, then stained for cell surface markers: CD8, CD122, and PD-1 to detect CD8+CD122+PD-1+ Tregs **(A)**. To quantify CD4+FoxP3+ Tregs, CD4 surface marker was first stained, and then intracellular marker FoxP3 was done **(B)**. PD-1 histogram was gated on CD8+CD122+ cells. One representative of three separate FACS data is shown (*p<0.05 and **p<0.01).

We next examined if GAG could ameliorate T1DM by up-regulating Tregs, especially in the presence of CsA, which has been shown to suppress CD4+FoxP3+ Treg development. Spleen and lymph node cells were isolated from female NOD mice following GAG and/or CsA treatments, and the percentage of CD4+FoxP3+ and CD8+CD122+PD-1+ Tregs was determined by FACS analyses eight weeks after treatments. As shown in Figure 2A, GAG significantly increased the percentage of CD8+CD122+PD-1+ Tregs in both lymph nodes and spleens of NOD mice in the absence or presence of CsA when compared with CsA alone or Model control. Moreover, as shown in Figure 2B, GAG also increased the percentage of CD4+FoxP3+ Tregs in both lymph nodes and spleens of NOD mice compared to Model group. As expected, CsA significantly reduced their percentage in both locations. Importantly, GAG was able to increase CD4+FoxP3+ Tregs even in the presence of CsA, suggesting a perfect synergy between GAG and CsA.

### GAG reduces the number of effector T cells (T_eff_) in NOD mice

**Figure 3:**
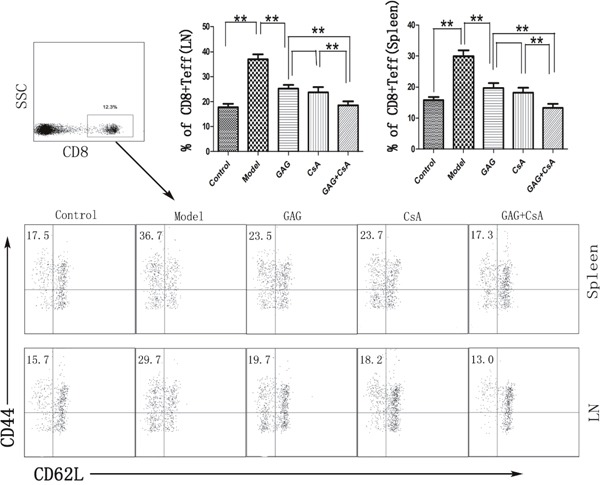
GAG shrinks effector CD8+ T cell (T_eff_) pool in NOD mice Spleen and lymph node cells were isolated from female NOD mice that were treated with GAG and/or CsA for eight weeks. Cells were then stained for CD8, CD44, CD62L surface markers. The dot plots for CD44 and CD62L were gated on CD8+ subset. One representative of three separate FACS data is shown (*p<0.05 and **p<0.01).

Since GAG was able to attenuate T1DM in NOD mice, we measured if GAG would influence the effector T cell differentiation. Spleen cells and lymph node cells derived from GAG-treated NOD mice were stained for surface markers CD8, CD44 and CD62L, and the percentage of CD8+CD44+CD62L^low^ cells was determined by FACS analyses eight weeks post-treatment. As shown in Figure 3, GAG significantly decreased the proportion of CD8+CD44+CD62L^low^ cells. In particular, treatments with both GAG and CsA further reduced the percentage of CD8+CD44+CD62L^low^ cells compared to GAG or CsA alone, indicating their synergistically suppressive effects on autoimmune T1DM.

### GAG hinders the infiltration of T lymphocytes in pancreas and alleviates the damage of pancreatic islet cells

**Figure 4:**
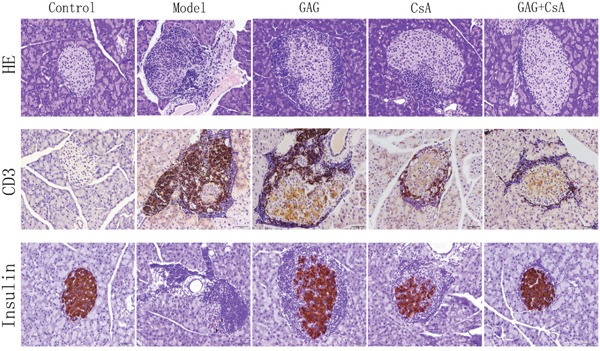
GAG and CsA significantly alleviate the infiltration of CD3+ T cells and general cellular infiltration in pancreatic islets Pancreases of NOD mice were collected eight weeks after treatments with GAG and/or CsA, and fixed in 4% neutral formaldehyde. Then paraffin-embedded tissues were stained with hematoxylin and eosin (HE) as well as anti-CD3 and anti-insulin mAbs for IHC. One representative of two sets of imaging is shown (Amplification: x200).

To visulize cellular infiltration and insulin expression in pancreatic tissues, we isolated the pancreases of NOD mice after eight weeks of treatments with GAG and/or CsA, and fixed them in 4% neutral formaldehyde. HE staining and immunohistochemical staining of CD3 and insulin were performed on paraffin-embedded tissue sections. As shown in Figure 4, the cellular infiltration, as shown by HE staining, in the group of GAG was obviously weakened compared to the Model group while CsA also reduced the cellular infiltration. GAG plus CsA further attenuated the cellular infiltration. Similarly, either GAG or CsA reduced the numbers of CD3+ T cells while GAG plus CsA further decreased their numbers. However, GAG or CsA alone increased insulin expression in pancreatic islets compared to the Model group while GAG plus CsA further enhanced the expression of insulin, suggesting that GAG or CsA alleviates the functional damage of islet cells.

### GAG significantly increases IL-10 but decreases IL-17 level in the content of pancreases of NOD mice

**Figure 5:**
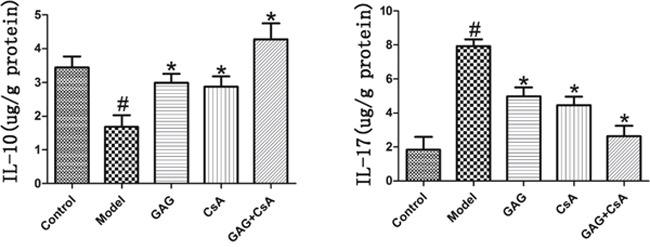
GAG up-regulates the level of IL-10 but down-regulates the level of IL-17 After eight weeks of treatments of NOD mice with GAG and/or CsA, pancreatic tissue was homogenized and the supernatant was collected to detect two cytokines IL-10 and IL-17 via ELISA. We utilized μg/g (protein) as the unit to express its content in the tissue. One representative of two separate experiments is shown. (# represents comparison with Control; and * represents comparison with Model, both p<0.05).

Since GAG was anti-inflammatory and could alleviate cellular infiltration in pancreases, we finally asked if GAG could exert its effects via regulating cytokines such as IL-10 and IL-17. After eight weeks of treatments with GAG and/or CsA, pancreatic tissue was homogenized and the supernatant was collected to detect these two cytokines by ELISA. As shown in Figure 5, the level of pro-inflammatory cytokine IL-17 was significantly reduced by GAG or CsA, especially when the combination of GAG and CsA was administered, while anti-inflammatory cytokine IL-10 was significantly increased by GAG and/or CsA. These findings suggest that GAG regulates autoimmunity and indeed is immunosuppressive.

## DISCUSSION

Using T1DM-prone NOD mice as readout to observe suppression of autoimmunity by Chinese medicine, we examined the effects of Chinese herbal formula GAG on the onset of T1DM in female NOD mice. We demonstrated that administration of GAG decreased the level of blood glucose but elevated the blood insulin level in NOD mice over time. Importantly, administering both GAG and low doses of CsA further ameliorated autoimmune T1DM compared with treatments with either GAG alone or CsA alone. Furthermore, GAG augmented both CD4+FoxP3+ and CD8+CD122+PD1+ Tregs in spleens and lymph nodes of NOD mice. In particular, GAG increased CD4+FoxP3+ Tregs even in the presence of CsA, which otherwise would hinder their generation. GAG also increased IL-10 but lowered IL-17 level in the content of pancreatic tissues of NOD mice. This finding may have clinical implications for the treatment of T1DM, especially in the early stage of the disease.

Conventional immunosuppressive agents, including CsA, may suppress autoimmunity and alleviate autoimmune diseases when administered during the early stage of the disease. However, patients with autoimmune diseases need continuous treatments with a conventional immunosuppressant that usually causes various side effects. Generally speaking, traditional Chinese medicine is believed to cause only moderate side effects. Ginseng and Astragalus granule (GAG), largely composed of Ginseng and Astragalus, has been widely used to treat type 2 diabetes mellitus in China [9, 10]. Previous studies have also demonstrated that GAG significantly ameliorates hyperglycemia, increases blood insulin level, and improves the glucose tolerance in animals with type 2 diabetes [11]. Here we found that GAG could also ameliorate autoimmune T1DM in NOD mice, which may lay the groundwork for clinical trials using GAG to treat T1DM in humans.

CD4+CD25+ regulatory T (Treg) cells play a key role in the maintenance of immune tolerance to both self- and foreign-antigens by suppressing aggressive T cell responses. These Tregs represent a small fraction (5-10%) of CD4+ T cells and constitutively express the α chain of the IL-2 receptor (CD25) [12–14]. The induction of endogenous CD4+CD25+ Tregs or adoptive transfer of exogenous Tregs prevents autoimmune diseases and allograft rejection in many animal models [15–22]. Recently, mounting evidence has demonstrated that CD8+CD122+ T cells are also Tregs that inhibit conventional T cell responses [23–28], antitumor immunity [29], as well as autoimmunity [30, 31]. We have previously shown that CD8+CD122+ T cells are not only Tregs [32, 33], but also are more potent in suppression of allograft rejection than conventional CD4+CD25+ Tregs [34]. In particular, we previously have demonstrated that PD-1-positive component within CD8+CD122+ T cell population is mainly responsible for their regulatory activities while antigen-specific CD8+CD122+PD-1- T cells are true memory T cells [32]. Therefore, CD8+CD122+ Tregs likely correspond to their CD4+CD25+ counterparts since CD122 is the β subunit of IL-2 receptor on T cells while CD25 is the α subunit of the same receptor [12], suggesting that both subsets of Tregs may cooperate to maintain the immunologic homeostasis and to keep autoimmune responses in check.

In this study, we found that GAG augmented both CD4+FoxP3+ and CD8+CD122+PD1+ Tregs in T1DM-prone NOD mice. Since GAG simultaneously increased both CD4+FoxP3+ and CD8+CD122+PD1+ Tregs, these two subsets of Tregs likely cooperated to exert their suppressive effects [28]. In particular, GAG increased traditional CD4+FoxP3+ Tregs even in the presence of CsA, indicating a much-needed synergy between GAG and CsA. Previous studies have shown that CsA is detrimental to the survival and function of Tregs via impeding the expression of IL-2 [35–37], which is crucial to Treg development. Moreover, CsA can suppress both generation and function of CD4+CD25+FoxP3+ Tregs in both mice and human beings [38–41]. Although CsA hinders generation and function of Tregs, it is still widely used to effectively treat patients with many of the autoimmune diseases. Therefore, combination of CsA and GAG is an ideal approach to maintaining the capacity of CsA to suppress autoimmune responses while sparing Tregs by GAG treatments. Our findings will establish a good example for treating autoimmune diseases using joint treatments with traditional Chinese medicine and Western medicine.

In conclusion, traditional Chinese medicine GAG, which effectively treats type 2 diabetes in clinic, can also alleviate autoimmune T1DM in NOD mice by upregulating both CD4+FoxP3+ and CD8+CD122+PD1+ Tregs. It also plays a regulatory role in controlling autoimmune responses by reducing IL-17 production in the pancreatic tissue given that IL-17 is critically involved in autoimmunity [42, 43]. Moreover, GAG regulates autoimmunity by increasing immunosuppressive cytokine IL-10. Since it increases both CD4+FoxP3+ and CD8+CD122+PD1+ Tregs as well as IL-10 production, an increase in IL-10 level is likely attributed to augmentation in both Tregs that in turn produce IL-10. Thus, in addition to type 2 diabetes, T1DM may also be treated using traditional Chinese medicine GAG. Our results have clinical implications.

## MATERIALS AND METHODS

### Mice and reagents

NOD mice were purchased from Beijing HFK Bioscience CO., LTD (Beijing, China). All mice were housed in a specific pathogen-free environment and all animal experiments were approved by the Institutional Animal Care and Use Committee. Traditional Chinese medicine formula, Ginseng and Astragalus granule (GAG), composed of Ginsenosides of it's stem and leaves, Schisandra, Astragalus, Yam, Radix rehmanniae, Ophiopogon japonicus, Poria, Radix trichosanthis, Rhizoma alismatis and Chinese wolfberry, was purchased from Lunan Houpu Pharmaceutical Co. Ltd (Lunan, China). Cyclosporin A (CsA) was purchased from the Guangdong Provincial Hospital of Traditional Chinese Medicine (Guangzhou, China). Anti-CD4-PE, anti-Foxp3-APC, anti-CD8-PE, anti-CD44-PE-Cy5, anti-CD62L-FITC, anti-CD8-FITC, anti-CD122-PE, and anti-PD-1-APC mAbs were purchased from BD Biosciences (BD Biosciences, San Jose, CA) or eBioscience (eBioscience, San Diego, CA).

### Treatments of mice

Female NOD mice were housed in a specific pathogen-free environment. When they were three months old, their random blood glucose levels were detected using Opitima Xceed and Test Strips (Abbott) once a week. GAG was dissolved in normal saline and orally administered at 3 g/kg/day to three-months-old NOD mice daily for eight weeks. Meanwhile, cyclosporin A was also diluted in normal saline and administered i.p. at 7.5 mg/kg/day while normal saline was used as controls (Model). After eight weeks' treatments, we continued to observe their condition and monitor their blood glucose etc. for two additional weeks. In addition to control groups treated with normal saline (Model group) versus GAG (GAG group), at the end of treatments when samples were collected, we used three-months old healthy NOD mice without any treatment as normal controls.

### Intracellular staining and flow analyses

Draining lymph nodes and spleen cells were isolated four and eight weeks after treatments. First, cells were stained for surface markers with anti-CD4-PE, anti-CD8-PE, anti-CD44-PE-Cy5, anti-CD62L-FITC anti-CD8-FITC, anti-CD122-FITC, or anti-PD-1-APC, separately in some samples, and then intracellular markers in some groups with anti-FoxP3-APC using intracellular fixation/permeabilization kit (eBioscience, San Diego, CA). CD4+Foxp3+ Tregs, CD8+CD122+PD-1+Tregs, and effecter T cells were enumerated by FACS analyses.

### Measurement of insulin and cytokines

Peripheral blood was collected and stored at room temperature for 30 minutes, then centrifuged at 3000xg for 20 minutes to collect the serum, and the insulin was detected using the ELISA kit (CUSABIO, Wuhan, China) according to the manufacturer's instructions. As for the interleukin-10 (IL-10) and interleukin-17 (IL-17), about 50 mg tissue was homogenized in 500ul precooled PBS to produce the mixture, which was then centrifuged at 5000xg at 4°C for 5 minutes. The supernatant was collected, and then we detected the total protein of the supernatant by Pierce^TM^ BCA Assaay Kit (Thermo Fisher Scientific, Massachusetts, USA). Finally, IL-10 and IL-17 in the supernatant were detected by ELISA kits (BOSTER, Wuhan, China). The unit of μg/g (protein) was utilized to express the content of both cytokines in the tissue.

### Hematoxylin-Eosin (HE) and Immunohistochemical (IHC) staining

Pancreatic tissues were fixed in 4% neutral formaldehyde for 24-48 hours, and embedded in paraffin, then cut into 3-4 μm sections. The sections were dried at 55°C, then deparaffinized in Xylen, followed by dehydration through the graded alcohol. Some tissue sections were only stained with hematoxylin and eosin. Moreover, endogenous peroxidase activity was blocked by 3% H_2_0_2_. The sections were then incubated with primary monoclonal anti-CD3 and anti-insulin antibodies (Abcam, Cambridge, UK) at 4°C over night. After incubation with HRP-anti-mouse IgG, sections were colored using 3′3′-diaminobenzidene (DAB, Sigma-Aldrich) and counterstained by Hematoxylin.

### Statistical analysis

Comparisons of the mean were performed using the Student *t-test* for two groups and ANOVA for multiple groups. All analyses were performed using Prism-6 software (GraphPad Software, La Jolla, CA). Data were presented as Mean ± SD. A value of P<0.05 was considered statistically significant.

## Abbreviations

CsA: cyclosporine
GAG: ginseng and astragalus granule
T1DM: type 1 diabetes mellitus
Treg: regulatory T cell
